# Matrix Metalloproteinase 9 in Epilepsy: The Role of Neuroinflammation in Seizure Development

**DOI:** 10.1155/2016/7369020

**Published:** 2016-12-26

**Authors:** Elżbieta Bronisz, Iwona Kurkowska-Jastrzębska

**Affiliations:** 2nd Department of Neurology, Institute of Psychiatry and Neurology, Jana III Sobieskiego 9, 02-957 Warsaw, Poland

## Abstract

Matrix metalloproteinase 9 is a proteolytic enzyme which is recently one of the more often studied biomarkers. Its possible use as a biomarker of neuronal damage in stroke, heart diseases, tumors, multiple sclerosis, and epilepsy is being widely indicated. In epilepsy, MMP-9 is suggested to play a role in epileptic focus formation and in the stimulation of seizures. The increase of MMP-9 activity in the epileptic focus was observed both in animal models and in clinical studies. MMP-9 contributes to formation of epileptic focus, for example, by remodeling of synapses. Its proteolytic action on the elements of blood-brain barrier and activation of chemotactic processes facilitates accumulation of inflammatory cells and induces seizures. Also modification of glutamatergic transmission by MMP-9 is associated with seizures. In this review we will try to recapitulate the results of previous studies about MMP-9 in terms of its association with epilepsy. We will discuss the mechanisms of its actions and present the results revealed in animal models and clinical studies. We will also provide a comparison of the results of various studies on MMP-9 levels in the context of its possible use as a biomarker of the activity of epilepsy.

## 1. Introduction

Discovered in 1974 matrix metalloproteinase 9 (MMP-9), also known as gelatinase B, belongs to the superfamily of proteolytic enzymes, degrading extracellular matrix and influencing almost all aspects of mammalian cell biology. The characteristic structure of this group of enzymes is based on a prodomain and a catalytic domain containing zinc, to which other domains distinguishing different families and enzymes are attached. Belonging to the family of gelatinases MMP-9 is one of the most complex matrix metalloproteinases. In its structure, apart from the prodomain and the catalytic domain, a fibronectin-like, a hemopexin, and a type V collagen-like domain are included [1]. Thanks to its complex structure MMP-9 is able to bind with various substrates for example, tissue inhibitors of metalloproteinases (TIMPs), laminin, gelatin, collagen types I and IV, procollagen type II, *β*-amyloid (1–40), precursors of growth factors, chemokines, surface receptors, and adhesion molecules (e.g., [1, 2]). According to proteomic research the number of possible substrates of MMP-9 may reach even a few hundred. Their variety is associated with diverse activities of MMP-9 within the central nervous system (CNS).

The presence of MMP-9 was shown within the hippocampus, cerebral cortex, and cerebellum [3]. The expression of MMP-9 was observed in neural and glial cells [4]: above all in astrocytes and microglia [5]. Within the cell the presence of the active form of MMP-9 was confirmed in nucleus of neurons and glia [6, 7]. In the synapses the presence of MMP-9 mRNA, protein, and enzymatic activity was shown in dendritic spines containing the postsynaptic part of excitatory synapses [4, 8–10]. Moreover, inflowing leukocytes are the source of MMP-9 in CNS [11]. MMP-9 is also secreted by endothelial cells [12]. Small amount of protease is released constantly, though its level and activity rise significantly after various stimuli, both physiological and pathological [13].

The secretion of MMP-9 is regulated on many levels. Gene expression for MMP-9 within the brain is dependent mainly on two transcription factors: activator protein 1 (AP-1) and nuclear factor-*κ*B (NF-*κ*B). An increase of MMP-9 expression in neurons is induced by their depolarization and activation of receptors [14, 15]. In microglial cells, inflammatory factors cause the increase of MMP-9 expression [16, 17]. Transcription is influenced by various cytokines, chemokines and growth factors. In human neural cells interleukin- (IL-) 1*β* increases transcription of MMP-9 and transforming growth factor *β* (TGF-*β*) inhibits it [18]. Nerve growth factor (NGF) and brain-derived neurotrophic factor (BDNF) increase expression and activity of MMP-9 [19, 20].

MMP-9 is secreted from neurons (dendrites) under the influence of glutamate [21]. MMP-9 is released in an inactive form. The activation of MMP-9 is reached in two ways. The first is cleaving of the propeptide. This role may be fulfilled by different metalloproteinases—mainly MMP-3—and components of plasminogen-plasmin cascades. The other way of activation is lysis of cysteine residue [22]. The activity of MMP-9 is inhibited by endogenous proteins TIMPs. Among them TIMP-1 is considered to be a natural inhibitor of MMP-9. The misbalance between MMP-9 and TIMP is associated with numerous CNS diseases [23]. MMP-9 exerts its effects mostly in the extracellular space [3]. However, possibility of its actions inside the cells was also described (e.g., [24]; for molecular biology of MMP-9, see [25]).

According to recent studies, MMP-9 seems to be a key factor in pathogenesis of epilepsy. It is assumed that impaired plasticity within the synapse and mossy fibers sprouting within the hippocampus, both associated with MMP-9 activity, have an impact on the formation of a new epileptic focus [26–28]. The other kind of process interrelated with epileptogenesis is inflammatory processes within CNS, whose regulation is also influenced by MMP-9 activity. Additionally, MMP-9 is associated with blood-brain barrier damage which lowers seizure threshold and induces formation of new epileptic foci [29, 30]. In next paragraphs the effects of MMP-9 activity and its associations with epilepsy will be presented.

## 2. Effects of MMP-9 Activity and Epilepsy

### 2.1. Extracellular Matrix

One of the activities of MMP-9 is lysis of the extracellular matrix (ECM). Noteworthy, ECM is not only a passive scaffolding on which other functional elements are deployed. The main components of ECM are proteoglycans, hyaluronic acid, link proteins, and tenascins. Proteoglycans mainly belong to the family of hyalectans binding with hyaluronic acid in macromolecular complexes with help of link proteins [31]. So far its inhibitory effect on axon growth in in vitro research has been reported (e.g., [32]). Proteoglycans also exert a plasticity-limiting effect in CNS and change the ECM composition to characteristic for adults [33]. Tenascins are macromolecular glycoproteins able to bind with adhesion molecules and surface receptors [31]. Apart from these main elements, ECM comprises, for example, laminin, thrombospondin, lectins, and matrix metalloproteinases.

The components of ECM participate in regulation of ion homeostasis, which is being suggested for example, on the basis of initial axonal segment formation on the foundation of ECM proteins [34]. Recently also the questions of probable neuroprotective activity of perineuronal nets (PNN) are being taken into concern. The PNN are big aggregates of ECM, surrounding subpopulations of neurons, which according to recent data exert neuroprotective effect in neurotoxicity caused by amyloid-*β*. Their neuroprotective activity against oxidative stress has also been suggested [35]. ECM is also said to be the forth part of the synapse (apart from the pre- and postsynaptic part of neurons and a glial cell) which modulates activity of ion channels and receptors within the synapse [36]. Among the components of ECM influencing synaptic changes and in consequence formation of new epileptic foci, MMP-9 attracts much attention [37].

Changes within the synapse associated with epilepsy are exerted in dendritic spines [38]. Modification of their morphology is linked with MMP-9 activity [39]. Michaluk et al. [39] noted that in neural cells of the hippocampus chronic enzymatic activity of MMP-9 causes elongation and thinning of dendritic spines. Temporary increase of the MMP-9 level on the other hand induces their growth and change of shape to a mushroom-like [40]. The presence of MMP-9 is essential for dendritic spines growth [41]. The change of dendritic spines' morphology is caused by MMP-9 activity and is mediated by *β*-dystroglycan, intercellular adhesion molecule 5 (ICAM-5), and *β*1, *β*2, and *β*4 integrins [42–48]. After cleaving of neuroligin 1 by MMP-9 modification of synaptic transmission was observed. The modification was associated with a change in the presynaptic part [49]. Another substrate of MMP-9 responsible for changes in synapse structure and activity is synaptic cell adhesion molecule-2 (synCAM-2) [50]. Additionally, MMP-9 cleaves a postsynaptically localized protein, nectin-3 [51], and proteins responsible mainly for intrasynaptic transmission: *β*-amyloid peptide [52], insulin-like growth factor-binding proteins [53], and IL-1*β* [54]. Also the change of the structure of dendritic spines may influence transmission within the synapse.

MMP-9 affects induction of long-term potentiation (LTP), a main model of synaptic plasticity, changing simultaneously structure and functioning of the synapse [40]. As is noted by Matsuzaki et al. [55] the induction of LTP is associated with enlargement and remodeling of small dendritic spines presenting only N-methyl-D-aspartate (NMDA) receptors to big synapses presenting both NMDA and *α*-amino-3-hydroxy-5-methyl-4-isoxazolepropionic acid (AMPA) receptors. The activity of NMDA receptors in turn modifies the levels and activity of MMP-9 what may be a significant step in generating LTP [56]. After the seizures the activity of MMP-9 associated with AMPA and NMDA receptors increases [9]. MMP-9 causes degradation of amyloid precursor protein what influences the secretion of soluble amyloid precursor protein *α*, associated with neuronal plasticity and memorization, and LTP within the hippocampus [57]. Noteworthy, MMP-9 affects both LTP induction [40] and its maintenance [58]. In MMP-9 knockout mice a dysfunction of the late phase of LTP was noted [59], similarly as it was reported in animals in which MMP-9 was inhibited by TIMP-1 or a MMP-9 specific inhibitor [60]. The influence of MMP-9 on LTP is still being analyzed; however, it has to be noted that, by regulation of LTP, MMP-9 modifies processes of learning and memory. Research carried out so far indicate that MMP-9 plays a vital role in spatial and appetitive memory. The inhibition of MMP-9 leads to LTP abnormalities within the hippocampus and in consequence influences memorizing of localization in space [59]. The appetitive memory is associated with the expectation of reward and development of addiction. The activity of MMP-9 is increased within the central nucleus of the amygdala and is essential to memorizing associated with the expectation of reward [61].

### 2.2. Other Functions

MMP-9 takes part in many physiological processes: cell differentiation and migration, tissue healing and remodeling, cytokine secretion, regulation of growth factors activity, and regulation between pro- and anti-inflammatory processes and between the processes of survival and apoptosis [62–64]. So far the role of MMP-9 has been reported, for example, in oligodendrocyte differentiation [65], Schwann cell migration [66], neuronal regrowth [67], degradation of active NGF [68], or conversion of pro-BDNF [4]. In MMP-9 knockout mice a delay of migration and a decrease of apoptosis of cerebellar granule cells were reported [69]. Some of the aforementioned effects, that is, Schwann cell migration and neuronal regrowth, are the effects of nonenzymatic activity of MMP-9.

MMP-9 participates also in many pathological processes. Among others its role was noted in cancerogenesis and metastasis [70], pathology of learning and memory, addiction, and psychiatric diseases (schizophrenia, bipolar disorder, and cognitive disorders) as well as in inflammatory and neurodegenerative diseases, multiple sclerosis, and cerebral ischemia [22].

### 2.3. Epileptic Focus

In the context of epilepsy the effect of MMP-9 was noted in tissue remodeling within the epileptic focus after the occurrence of seizures. In the study of Szklarczyk et al. [71] an association between remodeling within dentate gyri and an increased expression and activity of MMP-9 was reported after the kainate-induced seizures in rats. A greater susceptibility to epileptogenic process was observed in transgenic rats which exhibited overexpression of the active form of MMP-9 in the kindling model induced by pentylenetetrazole [8]. Also other researchers noted the association of MMP-9 with reorganization of neuronal circuits [4]. Impaired synaptic plasticity is essentially linked with epilepsy [72]. During kindling, which is a repeated brain stimulation, it comes to lowering of the seizure threshold and eventually to spontaneous occurrence of epileptic seizures. On the cellular level mossy fiber sprouting and formation of new synaptic connections are then being observed [73].

### 2.4. Inflammation

The other effect associated with epilepsy is the activation of inflammation. Numerous studies uncover an increase of the level or activity of MMP-9 after the induction by inflammatory factors (e.g., [16, 74]). MMP-9 affects inflammatory processes on many levels; for example, it enables leukocyte influx to the locus of inflammation through the blood-brain barrier, helps to form the chemotactic signal by releasing the chemokines from ECM, and activates tumor necrosis factor *α* (TNF-*α*) [75, 76]. Recently its double role in the regulation of pro- and anti-inflammatory processes has been noted. An example of that role is not only intensification, but also inhibition of chemotaxis by MMP-9 [77]. Lo et al. [78] note that the activity of MMP-9 in acute inflammation may be an attempt of rescuing the healthy tissues. Additionally, it was observed that factors that are said to be proinflammatory not always intensify the epileptic process; active microglia may also exert protective function during status epilepticus [79]. It seems that MMP-9 may be partially responsible for the maintenance of homeostasis between protection and damage.

When it comes to the prevalence of inflammatory processes a new epileptic focus is being formed. In the inflamed tissue microglia is being activated and astrocytes become reactive. The presence of reactive astrocytes was observed in epileptic foci in temporal epilepsy, focal cortical dysplasia, or tuberous sclerosis [80, 81]. These cells produce proinflammatory cytokines (e.g., IL-1*β*, IL-6, and TNF-*α*) responsible for the promotion of cell death after status epilepticus [82]. Moreover they secrete chemokines, recruiting inflammatory cells, for example, C-C motif chemokine ligands 2, 3, and 5 [83] what leads to further intensification of inflammatory processes. MMP-9 takes part in activation and deactivation of many chemokines and cytokines participating in inflammation, for example, IL-1*β*, TNF-*α*, and TGF-*β* [12, 84, 85].

MMP-9 plays a vital role in blood-brain barrier damage. The increase of blood-brain barrier permeability is one of the first abnormalities which occur in status epilepticus [30]. On the other hand, malfunctioning of blood-brain barrier causes lowering of seizure threshold independently of the cause of disruption [29], contributes to induction of epileptic discharges, and increases their frequency [86]. MMP-9 is a main factor participating in blood-brain barrier damage independently of the damaging factor [87]. The role of MMP-9 in blood-brain barrier disruption was reported in cerebral ischemia and inflammation [88–90].

MMP-9 causes damage to blood-brain barrier by cleaving zonula occludens 1 protein, one of the proteins regulating efficiency of tight junctions [91]. Moreover, collagen type IV, a main component of the basal lamina of endothelium, is a substrate for MMP-9 [11]. Opposing results concern occludin, a building block of tight junction connections, which is another protein on which the tightness of blood-brain barrier is dependent. In the research of Asahi et al. [91] MMP-9 influence on the occludin level was not confirmed, what on the contrary was observed by Reijerkerk et al. [92] and Zozulya et al. [93].

Released from the leukocytes during inflammation MMP-9 may be a factor promoting the inflammatory process by influencing blood-brain barrier permeability and enabling further influx of the cells participating in inflammation [12]. The role of tissue invading leukocytes in epileptic process comes to attention of, for example, Fabene et al. [94]. The enzymatic activity of metalloproteinases is vital for leukocyte migration [95].

Together with MMP-2, MMP-9 selectively cleaves dystroglycan, a protein fixing astrocyte endfeet to the basement membrane and thus allowing leukocyte infiltration to the brain parenchyma [96]. In genetically modified mice lacking both MMP-9 and MMP-2 Agrawal et al. observed resistance to the development of CNS inflammation as leukocytes had no ability to move from the perivascular space through the parenchymal basement membrane [96].

MMP-9 takes part both in chemotaxis and in migration of inflammatory cells. MMP-9 activates, for example, IL-8, granulocyte chemotactic protein, and many others [97, 98]. In a mouse model of meningitis crossing the endothelium by leukocytes contributes to further damaging of blood-brain barrier and induction of epileptic seizures [99]. Leukocytes biphasically influence formation of epileptic focus. In the acute phase they exacerbate the damage of blood-brain barrier. In the chronic phase they contribute to the release of cytokines, chemokines, and cytotoxic enzymes. They also cause changes in vessels and influence production of free oxygen radicals [100]. The permeability of blood-brain barrier has influence on dendritic cell migration as well. Produced by dendritic cells MMP-9 mediates their migration [93].

### 2.5. Glutamatergic Receptors

The effect of MMP-9 on glutamate receptors may also impact neuronal excitability and development of seizures. MMP-9 modifies both NMDA and AMPA receptors influencing the efficiency of glutamatergic transmission [43, 101]. MMP-9 increases the activity of NMDA receptor through integrins. After inhibition of integrin *β*1 with simultaneous presence of MMP-9 a total immobilization of NMDA receptor was observed [43]. The EphB receptor and *β*-dystroglycan, molecules affecting the activation of NMDA receptor, are substrates for MMP-9 [42, 102]. Also MMP-9 itself influences NMDA receptor causing its activation [59]. On the other hand, activation of NMDA receptor has influence on the expression and activity of MMP-9 [15] as well as on the modification of adhesion molecules and change of the morphology of dendritic spines [46]. MMP-9 cleaves an adhesion molecule ICAM-5 whose soluble extracellular domain affects the expression of AMPA receptor within the synapse [103]. Acting through the integrins MMP-9 decreases the activity of AMPA receptor [43]. As it has been shown so far, MMP-9 modifies glutamatergic transmission and influences the quantity of glutamate within the synapse.

### 2.6. Cell Death

**Figure 1:**
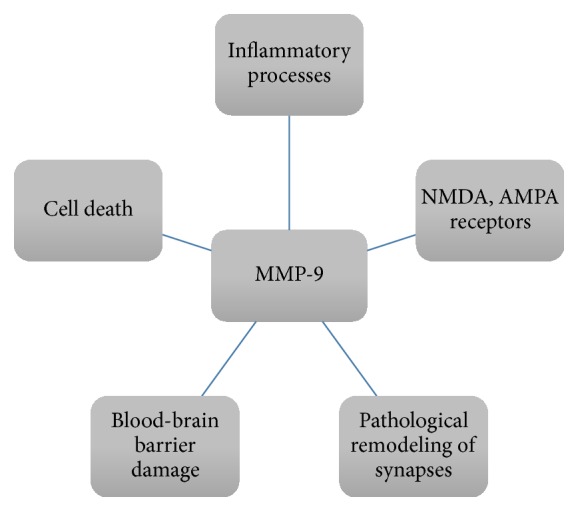
The fields of MMP-9 action in epilepsy.

It is being suggested that, in some models of epilepsy, MMP-9 contributes to cell death. In a kainate-induced seizure model MMP-9 causes cell death in the mechanism of excitotoxicity [104, 105]. In a model of status epilepticus induced by pilocarpine, Kim et al. reported cell death in a mechanism of apoptosis associated with MMP-9 activity [106]. Apart from epilepsy models MMP-9 was observed to cause cell death contributing, for example, to impairment of transmission between ECM and the cell through the lipoprotein receptor-related protein [107], separation of the cells from ECM leading to anoikis, the kind of apoptosis caused by the activation of proteolytic cascades [108], an increase of calpain activation [109], and initiation of caspase cascade and induction of cytotoxicity [77]. At a glance, the diversity of MMP-9 action in epilepsy is shown in Figure 1.

## 3. Animal Models

The relation of MMP-9 with the occurrence of epilepsy was confirmed in animal models: a kainate, a pilocarpine, a 4-aminopyridine, and a pentylenetetrazole model. Hippocampus, a region of great susceptibility to seizures and characterized by high probability of involvement in epileptic discharges [72], has been the most often studied structure. In the context of epilepsy attention has been paid mainly to cell damage associated with the occurrence of seizures, impairment of synapse plasticity, damage of blood-brain barrier, and inflammatory processes within CNS.

In a rat model of seizures induced by administration of kainic acid, an agonist of glutamatergic receptors, a correlation among the increase of the MMP-9 expression, activity and protein quantity, and remodeling within dentate gyri was reported [71]. Moreover an increase in TIMP-1 gene expression was observed. The results of the study suggested the influence of MMP-9 on pruning of dendritic spines and formation of abnormal synaptic connections [71]. In another rat kainate model after the occurrence of seizures the transportation of MMP-9 mRNA to the dendrites and synapses within dentate gyri of the hippocampus was observed [110]. Also Jourquin et al. [104] observed an increased release and enzymatic activity of MMP-9 after the administration of kainate in organotypic hippocampal cultures. The researchers noted as well a decrease in cell mortality after blocking of the MMP-9 activity. Jourquin et al. paid also attention to the involvement of excitotoxic and inflammatory processes in neural cell damage. Other researchers observed that, in the kainate model, a vital role is played also by the oxidative stress [111]. The occurrence of apoptosis associated with the MMP-9 activity was reported by Kim et al. [106]. In their study the administration of pilocarpine initiated status epilepticus in rats what was associated with overexpression of MMP-9 and a decrease of *β*1-integrin concentration and consequently with death of hippocampal cells in the mechanism of apoptosis [106]. In another pilocarpine model in infant rats, MMP-9 also caused cell death and its inhibition was associated with decreased damage [112]. Lower susceptibility to cell death after pilocarpine administration induced seizure was also reported in MMP-9 knockout mice. In previously mentioned studies MMP-9 was associated with the occurrence of damage within the brain.

On the contrary, according to Takács et al. [113] the enzymatic activity of MMP-9 is mainly associated not with cell damage, but with the plasticity within the synapse, a reorganization of both its morphology and functioning. After the injection of 4-aminopyridine, a potassium channel blocker, an increased activity of MMP-9 in the regions of cortex involved in the generalized epileptic process was observed. Moreover, in a genetic model of absence-like seizures of Wistar Glaxo Riswijk rats, higher levels of MMP-9 proenzyme were reported in comparison to control animals. In these rats the occurrence of epileptic discharges was also observed, mainly on the border of sleep and awakening. In both above-mentioned models, a correlation between an increased enzymatic activity of MMP-9 and occurrence of epileptic activity that did not cause cell death was reported [113]. In another kainate model in rats with chronic epilepsy, Zahn et al. [114] indicate less frequent occurrence of seizures in these rats after administration of 4-aminopyridine what also suggests the role of MMP-9 in brain plasticity. Similar results came from the study of Wilczynski et al. [8] who observed the decrease in dendritic spines pruning in genetically MMP-9 knockout mice after stimulation of seizures with pentylenetetrazole and kainate in comparison to healthy animals. MMP-9 knockout mice were less susceptible to kindling and presented with less pronounced epileptic seizures. In genetically modified rats showing overexpression of MMP-9 the researchers reported an increased tendency to epileptogenesis after the administration of pentylenetetrazole. The formation of abnormal synaptic connections by mossy fiber sprouting was reported to correlate with MMP-9 expression in rat organotypic hippocampal cultures. Also in MMP-9 knockout mice a decrease in mossy fiber sprouting after the pentylenetetrazole administration was observed when compared to control animals. The possible explanation of this MMP-9 activity is the lysis of the ECM surrounding neurons and affecting synapse plasticity by controlling the shape of dendritic processes [8]. This physiological function of MMP-9 may be distorted in the process of the formation of new epileptic foci associated with changes of synapse morphology and kindling.

In another pentylenetetrazole model in mice, an increased expression and enzymatic activity of MMP-9 within the hippocampus were reported what was accompanied by epileptic seizures [4]. In this study a decreased progression of kindling was reported in MMP-9 knockout animals in comparison to control group. The level of mossy fiber sprouting was also lower in transgenic mice. Additionally, MMP-9 caused conversion of pro-BDNF to mature BDNF in mice which received multiple injections of pentylenetetrazole. BDNF affects axonal branching and changes of synapse morphology and contributes to formation of epileptic focus [115]. However, Mizoguchi et al. did not observe the increase of MMP-9 expression and activity after the single episode of seizures. The researchers stated that these results suggest regulation of the levels of MMP-9 expression and activity by multiple activation of NMDA receptors [4]. It complies with the older research of Giorgi et al. [116] in which inhibition of NMDA receptors prevented seizures and the development of kindling after the administration of pentylenetetrazole. Mizoguchi et al. underline the necessity of multiple stimulation by seizure inducing substance, though in other studies [14, 42] and even a single dose of seizure inducing substance was associated with the increased levels of MMP-9 mRNA and enzymatic activity. Possibly these differences can be explained by different animals chosen for the research and different times of measurements. Even though the results of the study conducted by Mizoguchi et al. confirm the role of MMP-9 in neuronal plasticity. They also concentrate on the possible exacerbation of the pathology initiated by the kindling process by following changes in morphology and functioning of the synapses.

MMP-9 is suggested to contribute to the formation of hyperexcitable neuronal circuits. In one of the recent studies with MMP-9 knockout infant mice, an increase of the number of pyramidal neurons in the CA1 region of hippocampus and a decrease of the number and complexity of dendritic processes were reported. The aberrations of dendritic cell structure lasted until adulthood. More frequent spontaneous discharges were observed in adult transgenic mice. Also kainate-induced seizures lasted longer and were more severe in these mice [117].

Attention has also been paid to an association of MMP-9 with inflammatory processes and blood-brain barrier damage in the context of epilepsy. The administration of lipopolysaccharide to the brain of rats resulted in the increase of blood-brain barrier permeability and associated increase of MMP-9 level [118]. In the basal lamina of endothelial cells the destruction of collagen type IV was also observed. This suggested a key role of MMP-9 in blood-brain barrier damage [119]. An impaired function of blood-brain barrier may both initiate epileptogenesis and intensify present epileptic discharges. After its damage it comes to protein extravasation. The serum proteins activate astrocytes and affect the formation of new epileptic focus [120]. The blood-brain barrier damage in epilepsy might be confirmed by an increased level of S100B, the protein specific for CNS in patients with chronic epilepsy [121]. Another molecule involved in epileptogenesis is TGF- *β*. Pro-TGF-*β* is one of the substrates for MMP-9 [85]. MMP-9 activates pro-TGF-*β* to its active form. Active TGF-*β* participates in the increase of blood-brain barrier permeability what in turn leads to serum protein extravasation, astrocyte activation, and further intensification of inflammatory processes. The study of Fabene et al. emphasizes the importance of inflammation in epilepsy [94]. After the modification of endothelium-leukocyte interactions in a mouse model of epilepsy the researchers observed a decrease of frequency of the spontaneous epileptic seizures. These data were also confirmed in the model of hippocampal sclerosis [94].

It seems that the activity of MMP-9 in epilepsy is multidirectional. On one hand it has influence on inflammatory processes, blood-brain barrier damage, impairment of neurotransmission, formation of pathological synaptic connections, and epileptic foci as well as cell death. On the other hand it exerts protective role by inhibition of chemotaxis and rescuing of healthy tissue.

## 4. Clinical Studies

The main source of information about the cerebral structure morphology in patients with epilepsy is specimens excised during neurosurgical operations. The patients qualified for the operation most frequently are diagnosed with drug-resistant epilepsy which is defined as “failure of adequate trials of two tolerated, appropriately chosen and used antiepileptic drug schedules (whether as monotherapies or in combination) to achieve sustained seizure freedom” [122]. Other patients qualified for the neurosurgical procedures are diagnosed with complex epileptic syndromes, for example, Lennox-Gastaut syndrome, or suffering from other diseases in which epileptic seizures such as tuberous sclerosis or Sturge-Weber syndrome occur [123].

Approximately 73% of patients qualified for neurosurgical operation are diagnosed with temporal lobe epilepsy (TLE) [124]. TLE is frequently associated with selective atrophy of the hippocampus, the hippocampal sclerosis. The histopathological picture of TLE with coexisting hippocampal sclerosis comprises neuronal loss, abnormalities of morphology and function of various receptors and channels, activated neurogenesis and formation of new synaptic connections, gliosis, inflammatory processes, and blood-brain barrier damage [124]. However, independently of hippocampal sclerosis loss of neurons and changes of synapse organization is reported also in other epileptic foci [125, 126]. Additionally there appear neurons of changed morphology; for example, in the amygdala they are smaller and have less first-row dendritic spines while the total number of dendritic spines is increased [125].

The most frequent form of focal epilepsy is mesial temporal lobe epilepsy (MTLE) [127]. In patients with MTLE with coexisting hippocampal sclerosis the MMP-9 activity was reported to be increased within neural and glial cells within CA1 and CA2 regions off hippocampus, in the granule cell layer of dentate gyrus and in the neocortex [26]. Additionally, the researchers observed a correlation between the localization of TIMP-1 and the activity of MMP-9 in neocortex. The results of the study seem to be in favor of the association of MMP-9 with drug-resistant MTLE and coexisting hippocampal sclerosis. In another study in patients with MTLE associated with hippocampal sclerosis a decreased level of the collapsin response mediated protein-2 (CRMP-2) was reported [128]. CRMP-2 is a crucial protein for axonal regrowth and maintenance of neuronal polarity within the hippocampus. CRMP-2 is also a substrate for MMP-9 [50]. An increased activity of MMP-9 may be a cause of neuronal plasticity disorders and their degeneration may be mediated by the degradation of CRMP-2.

In TLE an increased expression of the proinflammatory cytokines IL-1*β* and NF-*κ*B was reported [129]. On the other hand the increased expression of these cytokines causes an increase of MMP-9 level. A similar rise in the proinflammatory cytokine level was also found in focal cortical dysplasia [130] and tuberous sclerosis [131], disorders associated with occurrence of seizures. The level of MMP-9 activity was increased within the epileptic foci of patients with focal cortical dysplasia and tuberous sclerosis as well [10, 132]. Interestingly, an increase of MMP-9 activity was also reported in the unchanged tissue of patients with epilepsy [10]. In the study of Konopka et al. the increase of MMP-9 level was mostly marked postsynaptically, within the dendritic spines. Moreover the activity of MMP-9 was reported in the localization corresponding with the localization of abnormal mossy fibers [10]. The authors noted that MMP-9 may exacerbate pathological processes occurring after seizures and lead to drug-resistant epilepsy.

## 5. MMP-9 as a Biomarker Associated with Epilepsy

In the research conducted so far an increased level of MMP-9 was reported in the blood serum of children who developed epileptic seizures. The increase of the MMP-9 level and the MMP-9 to TIMP-1 ratio was observed in patients with acute encephalopathy after febrile seizures, what was associated with blood-brain barrier damage [133]. Interestingly, Brew et al. [134] noted that the increase of MMP-9 serum level causes even greater increase of the level of TIMP-1. In children with human herpesvirus-6 (HHV-6) infection who developed seizures no significant difference in the level of MMP-9 activity was reported in comparison to infected children without coexisting seizures. In children with febrile seizures a decreased MMP-9 to TIMP-1 ratio was observed due to the TIMP-1 increase. Yet the HHV-6 infection itself caused an increase of serum MMP-9 level [135]. The authors suggested that MMP-9 level and MMP9/TIMP1 ratio might indicate central nervous system damage and development of encephalopathy.

In patients who developed epilepsy as a complication of acute encephalitis no discrepancies of MMP-9 levels were found between the experimental and the control group. However, the MMP-9 to TIMP-1 ratio was significantly increased in these patients, especially in the group with a history of encephalitis by the age of 6 years. Moreover the mean MMP-9 to TIMP-1 ratio was higher in patients with worse control of epilepsy (defined by authors as a minimum of 28 seizures during four weeks) than in the patients in whom lower number of seizures was observed. The mean levels of MMP-9 and TIMP-1 were similar in both groups [136]. The level of MMP-9 and MMP-9 to TIMP-1 ratio in blood serum was increased in children with influenza caused encephalopathy, both in the group with worse prognosis and in the group who developed febrile seizures. Even though the level of MMP-9 in these patients was comparable to the level of MMP-9 in patients with uncomplicated influenza, the patients with neurological complications had increased MMP-9 to TIMP-1 ratio in comparison to the patients without complications [137].

In patients with TLE with coexisting hippocampal sclerosis an increased activity of MMP-9, an elevated MMP-9 to TIMP-1 ratio and a high level of urokinase uPAR were reported. These patients were qualified for lobectomy. After the operation a decrease of serum MMP-9 and uPAR levels was observed. Moreover, during a year after the lobectomy these patients were free from seizures. The level of MMP-9 proenzyme and activity in the tissue of epileptic focus was increased in comparison to surrounding tissues and a normal hippocampus [138].

A specific group of patients with epilepsy are patients with neuropsychiatric manifestation of systemic lupus erythematosus (NPSLE). Among the patients with systemic lupus erythematosus (SLE), the group of patients in whom at least one neurological or psychiatric symptom, defining NPSLE, occurred, was reported to have increased level of MMP-9 in blood serum [139]. Among these patients four were diagnosed with epilepsy. However, the level of MMP-9 in patients with NPSLE manifesting as epilepsy was not higher than in patients with other neuropsychiatric diseases. It may result partially from underlying disease, severity of other symptoms (subjects were diagnosed among others with demyelinating syndrome or a polyneuropathy), or a small group of patients in the study.

In a varied group of patients we have recently shown a significant increase of serum MMP-9 in 1 hour and in 24 hours after seizures [140, 141]. The level of MMP-9 returned to control in 72 hours after seizures. The group of patients in the study consisted of patients with generalized tonic-clonic seizures, patients with convulsive and nonconvulsive status epilepticus, and patients with complex partial seizures. Apart from MMP-9, the levels of ICAM-1, E-selectin, and thrombomodulin were measured [140]. ICAM-1 was found to be increased only 1 hour after seizures and there were no differences in the levels of E-selectin and thrombomodulin in comparison to control group. The increase of MMP-9 and ICAM-1 was similar in all patients with epilepsy and correlated with the intensity of seizures. The results may suggest the activation of endothelium after seizures.

An increase of the MMP-9 level was found in cerebrospinal fluid of patients with bacterial meningitis who subsequently developed epileptic seizures in comparison to the patients in whom the disease was not complicated by the development of epilepsy [142]. The result of the study suggested the role of MMP-9 in blood-brain barrier damage what was confirmed by Li et al. [11]. In their study cerebrospinal fluid was collected from the patients with a history of generalized tonic-clonic epileptic seizure. In these patients an increased level of MMP-9 was reported in comparison to control group. The increase of MMP-9 concentration correlated to blood-brain barrier damage, an increase of the albumin quotient, and the number of leukocytes in cerebrospinal fluid. An increase of the albumin quotient reflects the leakage of serum proteins to the cerebrospinal fluid and hence the blood-brain barrier permeability. In aforementioned study the increase of the MMP-9 level in cerebrospinal fluid and associated increase of blood-brain barrier permeability were reported in the period of 24 hours from the occurrence of seizures. According to the authors an increased level of MMP-9 was a marker of the influx of activated leukocytes [11].

In one of the newer studies levels of matrix metalloproteinases (among them MMP-9), oxidative stress and LDH were checked in saliva of children with epilepsy. The patients were divided into two groups on the basis of disease control: a group with well-controlled seizures and a group in which the level of control was unsatisfactory. A decrease of MMP-9 level in the saliva of all patients with epilepsy was reported and it was most significant in the patients with the unsatisfactory control of the disease. A similar decrease was noted in the levels of LDH and MMP-3 while the level of MMP-2 remained stable in all groups of patients. Additionally, the level of oxidative stress was found to be increased in the children with epilepsy. The study, apart from noting the association of the decrease in MMP-9 level in saliva with the occurrence and activity of epilepsy, also shows a possibility of noninvasive evaluation of MMP-9 level [143].

The results of the studies on MMP-9 level in the above-mentioned groups of patients are shown in Table 1. Unfortunately, as for now we lack data regarding levels of MMP-9 in other groups of patients with epilepsy. However the research conducted so far suggests a possibility of evaluation of MMP-9 as a potential marker of blood-brain barrier damage which increased concentration is associated with occurrence of epileptic seizures.

## 6. Other Potential Uses

The concept of MMP-9 as a biomarker is not new. In stroke an increase of MMP-9 is regarded to be a marker of increased blood-brain barrier permeability and hemorrhagic transformation of ischemic foci [144]. An increase of MMP-9 has failed though as a single marker of stroke and yet a panel of biomarkers showed sensitivity and specificity on the level of approximately 90% [145, 146]. On the other hand, together with the increase of the level of natriuretic peptide type B and MMP-2 an increase of the MMP-9 concentration correlates to cardiac diseases [147]. Moreover an increased level of MMP-9 is a predictive factor of increased mortality due to stroke and impaired healing of ulcerations associated with diabetic foot, and a MMP-9 to TIMP-1 ratio together with the level of MMP-10 is tightly correlated to severity and mortality of sepsis [148]. In multiple sclerosis an increased level of MMP-9 in blood serum and cerebrospinal fluid is correlated to the course of disease. Probably it may also be a predictive factor of multiple sclerosis progression [149]. As far as neoplastic diseases are concerned high expression of MMP-9 in cancer cells is, for example, a negative prognostic factor of survival in esophageal cancer [150] whereas an increase of the level of MMP-9 in blood serum is associated with breast, pancreas, colon, and prostate cancer as well as an advanced lung cancer [151].

## 7. Conclusions

MMP-9 is one of the most widely met metalloproteinases in the brain [22]. Despite its broad distribution in the CNS and a huge contribution of many researchers in exploring this metalloproteinase, still many questions stay unresolved. Apart from its undoubted physiological role in the processes of cell differentiation, tissue remodeling, angiogenesis, regulation of the level and activity of cytokines and growth factors, regulation of pro- and anti-inflammatory processes, and processes leading to cell death or survival, MMP-9 also takes part in pathological processes. In epilepsy, both experimental research and clinical studies indicate that MMP-9 contributes to formation of epileptic focus, activation of inflammation processes after the occurrence of epileptic seizures via modification of blood-brain barrier and cell death. The association between MMP-9 with drug-resistance of epilepsy has also been noticed. Yet the knowledge about MMP-9 and its role in epilepsy needs further deepening.

## Figures and Tables

**Table 1:** The results of the studies on MMP-9 level in blood serum, cerebrospinal fluid, and saliva (indications: “↑” increase; “↓” decrease; “?” no information).

Disease entity	MMP-9	MMP-9/TIMP-1	Material	References
Encephalopathy after the occurrence of febrile seizures versus febrile seizures	↑↑	↑↑	Blood serum	Suenaga et al. [133]
Febrile seizures versus control	↑	↑	Blood serum	Suenaga et al. [133]
HHV-6 infection with coexisting febrile seizures versus HHV-6 infection without seizures	No influence	↓	Blood serum	Kittaka et al. [135]
HHV-6 infection without seizures versus control	↑	No influence	Blood serum	Kittaka et al. [135]
Epilepsy after acute encephalitis versus control	No influence	↑	Blood serum	Suriadi et al. [136]
Febrile seizures in children with encephalopathy caused by influenza versus control	↑	↑	Blood serum	Ichiyama et al. [137]
TLE with coexisting hippocampal sclerosis versus control	↑	↑	Blood serum	Quirico-Santos et al. [138]
TLE with coexisting hippocampal sclerosis versus patients after lobectomy due to hippocampal sclerosis	↑	↑	Blood serum	Quirico-Santos et al. [138]
Epilepsy in patients with NPSLE versus patients with SLE without the neuropsychiatric manifestation	↑	?	Blood serum	Ainiala et al. [139]
Patients after generalized tonic-clonic seizures, convulsive and nonconvulsive status epilepticus, and partial complex seizures versus control	↑	?	Blood serum	Cudna and Kurkowska-Jastrzębska [140], Cudna et al. [141]
Bacterial meningitis complicated by febrile seizures versus uncomplicated bacterial meningitis	↑	?	Cerebrospinal fluid	Leppert et al. [142]
Patients after generalized tonic-clonic seizure versus control	↑	?	Cerebrospinal fluid	Li et al. [11]
Epilepsy in children versus control	↓	?	Saliva	Shahar et al. [143]
Poorly controlled epilepsy in children versus well controlled epilepsy in children	↓↓	?	Saliva	Shahar et al. [143]

## Abbreviations

AMPA: *α*-Amino-3-hydroxy-5-methyl-4-isoxazolepropionic acid
AP-1: Activator protein 1
BDNF: Brain-derived neurotrophic factor
CNS: Central nervous system
CRMP-2: Collapsin response mediated protein-2
ECM: Extracellular matrix
HHV-6: Human herpesvirus-6
ICAM-5: Intercellular adhesion molecule 5
IL: Interleukin
LDH: D-Lactate dehydrogenase
LTP: Long-term potentiation
MMP: Matrix metalloproteinase
mRNA: Messenger ribonucleic acid
MTLE: Mesial temporal lobe epilepsy
NF-*κ*B: Nuclear factor-*κ*B
NGF: Nerve growth factor
NMDA: N-Methyl-D-aspartate
NPSLE: Neuropsychiatric manifestation of systemic lupus erythematosus
PNN: Perineuronal nets
SLE: Systemic lupus erythematosus
synCAM-2: Synaptic cell adhesion molecule-2
TGF-*β*: Transforming growth factor *β*
TNF-*α*: Tumor necrosis factor *α*
TLE: Temporal lobe epilepsy
TIMPs: Tissue inhibitors of metalloproteinases
uPAR: Urokinase receptor.

## References

[1] Van Den Steen P. E., Dubois B., Nelissen I., Rudd P. M., Dwek R. A., Opdenakker G. (2002). Biochemistry and molecular biology of gelatinase B or matrix metalloproteinase-9 (MMP-9). *Critical Reviews in Biochemistry and Molecular Biology*.

[2] Farina A. R., Mackay A. R. (2014). Gelatinase B/MMP-9 in tumour pathogenesis and progression. *Cancers*.

[3] Stawarski M., Stefaniuk M., Wlodarczyk J. (2014). Matrix metalloproteinase-9 involvement in the structural plasticity of dendritic spines. *Frontiers in Neuroanatomy*.

[4] Mizoguchi H., Nakade J., Tachibana M. (2011). Matrix metalloproteinase-9 contributes to kindled seizure development in pentylenetetrazole-treated mice by converting pro-BDNF to mature BDNF in the hippocampus. *Journal of Neuroscience*.

[5] Dzwonek J., Rylski M., Kaczmarek L. (2004). Matrix metalloproteinases and their endogenous inhibitors in neuronal physiology of the adult brain. *FEBS Letters*.

[6] Pirici D., Pirici I., Mogoanta L. (2012). Matrix metalloproteinase-9 expression in the nuclear compartment of neurons and glial cells in aging and stroke. *Neuropathology*.

[7] Sbai O., Ould-Yahoui A., Ferhat L. (2010). Differential vesicular distribution and trafficking of MMP-2, MMP-9, and their inhibitors in astrocytes. *GLIA*.

[8] Wilczynski G. M., Konopacki F. A., Wilczek E. (2008). Important role of matrix metalloproteinase 9 in epileptogenesis. *The Journal of Cell Biology*.

[9] Gawlak M., Górkiewicz T., Gorlewicz A., Konopacki F. A., Kaczmarek L., Wilczynski G. M. (2009). High resolution in situ zymography reveals matrix metalloproteinase activity at glutamatergic synapses. *Neuroscience*.

[10] Konopka A., Grajkowska W., Ziemiańska K. (2013). Matrix metalloproteinase-9 (MMP-9) in human intractable epilepsy caused by focal cortical dysplasia. *Epilepsy Research*.

[11] Li Y.-J., Wang Z.-H., Zhang B. (2013). Disruption of the blood-brain barrier after generalized tonic-clonic seizures correlates with cerebrospinal fluid MMP-9 levels. *Journal of Neuroinflammation*.

[12] Candelario-Jalil E., Yang Y., Rosenberg G. A. (2009). Diverse roles of matrix metalloproteinases and tissue inhibitors of metalloproteinases in neuroinflammation and cerebral ischemia. *Neuroscience*.

[13] Vafadari B., Salamian A., Kaczmarek L. (2016). MMP-9 in translation: from molecule to brain physiology, pathology, and therapy. *Journal of Neurochemistry*.

[14] Rylski M., Amborska R., Zybura K. (2009). JunB is a repressor of MMP-9 transcription in depolarized rat brain neurons. *Molecular and Cellular Neuroscience*.

[15] Meighan S. E., Meighan P. C., Choudhury P. (2006). Effects of extracellular matrix-degrading proteases matrix metalloproteinases 3 and 9 on spatial learning and synaptic plasticity. *Journal of Neurochemistry*.

[16] Woo M.-S., Park J.-S., Choi I.-Y., Kim W.-K., Kim H.-S. (2008). Inhibition of MMP-3 or -9 suppresses lipopolysaccharide-induced expression of proinflammatory cytokines and iNOS in microglia. *Journal of Neurochemistry*.

[17] Bartholomé E. J., Van Aelst I., Koyen E. (2001). Human monocyte-derived dendritic cells produce bioactive gelatinase B: inhibition by IFN-*β*. *Journal of Interferon and Cytokine Research*.

[18] Vecil G. G., Larsen P. H., Corley S. M. (2000). Interleukin-1 is a key regulator of matrix metalloproteinase-9 expression in human neurons in culture and following mouse brain trauma in vivo. *Journal of Neuroscience Research*.

[19] Kuzniewska B., Rejmak E., Malik A. R., Jaworski J., Kaczmarek L., Kalita K. (2013). Brain-derived neurotrophic factor induces matrix metalloproteinase 9 expression in neurons via the serum response factor/c-Fos pathway. *Molecular and Cellular Biology*.

[20] Muir D. (1994). Metalloproteinase-dependent neurite outgrowth within a synthetic extracellular matrix is induced by nerve growth factor. *Experimental Cell Research*.

[21] Michaluk P., Kaczmarek L. (2007). Matrix metalloproteinase-9 in glutamate-dependent adult brain function and dysfunction. *Cell Death and Differentiation*.

[22] Rivera S., Khrestchatisky M., Kaczmarek L., Rosenberg G. A., Jaworski D. M. (2010). Metzincin proteases and their inhibitors: foes or friends in nervous system physiology?. *Journal of Neuroscience*.

[23] Moore C. S., Crocker S. J. (2012). An alternate perspective on the roles of TIMPs and MMPs in pathology. *The American Journal of Pathology*.

[24] Wiera G., Wojtowicz T., Lebida K. (2012). Long term potentiation affects intracellular metalloproteinases activity in the mossy fiber-CA3 pathway. *Molecular and Cellular Neuroscience*.

[25] Vandooren J., Van Den Steen P. E., Opdenakker G. (2013). Biochemistry and molecular biology of gelatinase B or matrix metalloproteinase-9 (MMP-9): the next decade. *Critical Reviews in Biochemistry and Molecular Biology*.

[26] Acar G., Tanriover G., Acar F., Demir R. (2015). Increased expression of matrix metalloproteinase-9 in patients with temporal lobe epilepsy. *Turkish Neurosurgery*.

[27] Sharma A. K., Reams R. Y., Jordan W. H., Miller M. A., Thacker H. L., Snyder P. W. (2007). Mesial temporal lobe epilepsy: pathogenesis, induced rodent models and lesions. *Toxicologic Pathology*.

[28] Lukasiuk K., Wilczynski G. M., Kaczmarek L. (2011). Extracellular proteases in epilepsy. *Epilepsy Research*.

[29] Marchi N., Tierney W., Alexopoulos A. V., Puvenna V., Granata T., Janigro D. (2011). The etiological role of blood-brain barrier dysfunction in seizure disorders. *Cardiovascular Psychiatry and Neurology*.

[30] Gorter J. A., van Vliet E. A., Aronica E. (2015). Status epilepticus, blood-brain barrier disruption, inflammation, and epileptogenesis. *Epilepsy and Behavior*.

[31] Zimmermann D. R., Dours-Zimmermann M. T. (2008). Extracellular matrix of the central nervous system: from neglect to challenge. *Histochemistry and Cell Biology*.

[32] Yamaguchi Y. (2000). Lecticans: organizers of the brain extracellular matrix. *Cellular and Molecular Life Sciences*.

[33] Galtrey C. M., Fawcett J. W. (2007). The role of chondroitin sulfate proteoglycans in regeneration and plasticity in the central nervous system. *Brain Research Reviews*.

[34] Hedstrom K. L., Rasband M. N. (2006). Intrinsic and extrinsic determinants of ion channel localization in neurons. *Journal of Neurochemistry*.

[35] Suttkus A., Rohn S., Jäger C., Arendt T., Morawski M. (2012). Neuroprotection against iron-induced cell death by perineuronal nets—an in vivo analysis of oxidative stress. *American Journal of Neurodegenerative Disease*.

[36] Dityatev A., Rusakov D. A. (2011). Molecular signals of plasticity at the tetrapartite synapse. *Current Opinion in Neurobiology*.

[37] Huntley G. W. (2012). Synaptic circuit remodelling by matrix metalloproteinases in health and disease. *Nature Reviews Neuroscience*.

[38] Swann J. W., Al-Noori S., Jiang M., Lee C. L. (2000). Spine loss and other dendritic abnormalities in epilepsy. *Hippocampus*.

[39] Michaluk P., Wawrzyniak M., Alot P. (2011). Influence of matrix metalloproteinase MMP-9 on dendritic spine morphology. *Journal of Cell Science*.

[40] Wang X.-B., Bozdagi O., Nikitczuk J. S., Zhai Z. W., Zhou Q., Huntley G. W. (2008). Extracellular proteolysis by matrix metalloproteinase-9 drives dendritic spine enlargement and long-term potentiation coordinately. *Proceedings of the National Academy of Sciences of the United States of America*.

[41] Szepesi Z., Bijata M., Ruszczycki B., Kaczmarek L., Wlodarczyk J. (2013). Matrix metalloproteinases regulate the formation of dendritic spine head protrusions during chemically induced long-term potentiation. *PLoS ONE*.

[42] Michaluk P., Kolodziej L., Mioduszewska B. (2007). *β*-Dystroglycan as a target for MMP-9, in response to enhanced neuronal activity. *The Journal of Biological Chemistry*.

[43] Michaluk P., Mikasova L., Groc L., Frischknecht R., Choquet D., Kaczmarek L. (2009). Matrix metalloproteinase-9 controls NMDA receptor surface diffusion through integrin *β*1 signaling. *Journal of Neuroscience*.

[44] Court F. A., Zambroni D., Pavoni E. (2011). MMP2-9 cleavage of dystroglycan alters the size and molecular composition of Schwann cell domains. *Journal of Neuroscience*.

[45] Bijata M., Wlodarczyk J., Figiel I. (2015). Dystroglycan controls dendritic morphogenesis of hippocampal neurons in vitro. *Frontiers in Cellular Neuroscience*.

[46] Tian L., Stefanidakis M., Ning L. (2007). Activation of NMDA receptors promotes dendritic spine development through MMP-mediated ICAM-5 cleavage. *Journal of Cell Biology*.

[47] Vaisar T., Kassim S. Y., Gomez I. G. (2009). MMP-9 sheds the *β*2 integrin subunit (CD18) from macrophages. *Molecular and Cellular Proteomics*.

[48] Pal-Ghosh S., Blanco T., Tadvalkar G. (2011). MMP9 cleavage of the *β*4 integrin ectodomain leads to recurrent epithelial erosions in mice. *Journal of Cell Science*.

[49] Peixoto R. T., Kunz P. A., Kwon H. (2012). Transsynaptic signaling by activity-dependent cleavage of neuroligin-1. *Neuron*.

[50] Bajor M., Michaluk P., Gulyassy P., Kekesi A. K., Juhasz G., Kaczmarek L. (2012). Synaptic cell adhesion molecule-2 and collapsin response mediator protein-2 are novel members of the matrix metalloproteinase-9 degradome. *Journal of Neurochemistry*.

[51] Van Der Kooij M. A., Fantin M., Rejmak E. (2014). Role for MMP-9 in stress-induced downregulation of nectin-3 in hippocampal CA1 and associated behavioural alterations. *Nature Communications*.

[52] Ridnour L. A., Dhanapal S., Hoos M. (2012). Nitric oxide-mediated regulation of *β*-amyloid clearance via alterations of MMP-9/TIMP-1. *Journal of Neurochemistry*.

[53] Nishijima T., Piriz J., Duflot S. (2010). Neuronal activity drives localized blood-brain-barrier transport of serum insulin-like growth factor-I into the CNS. *Neuron*.

[54] Amantea D., Russo R., Gliozzi M. (2007). Early upregulation of matrix metalloproteinases following reperfusion triggers neuroinflammatory mediators in brain ischemia in rat. *International Review of Neurobiology*.

[55] Matsuzaki M., Honkura N., Ellis-Davies G. C. R., Kasai H. (2004). Structural basis of long-term potentiation in single dendritic spines. *Nature*.

[56] Ikonomidou C. (2014). Matrix metalloproteinases and epileptogenesis. *Molecular and Cellular Pediatrics*.

[57] Fragkouli A., Tzinia A. K., Charalampopoulos I., Gravanis A., Tsilibary E. C. (2011). Matrix metalloproteinase-9 participates in NGF-induced *α*-secretase cleavage of amyloid-*β* protein precursor in PC12 cells. *Journal of Alzheimer's Disease*.

[58] Bozdagi O., Nagy V., Kwei K. T., Huntley G. W. (2007). In vivo roles for matrix metalloproteinase-9 in mature hippocampal synaptic physiology and plasticity. *Journal of Neurophysiology*.

[59] Nagy V., Bozdagi O., Matynia A. (2006). Matrix metalloproteinase-9 is required for hippocampal late-phase long-term potentiation and memory. *Journal of Neuroscience*.

[60] Okulski P., Jay T. M., Jaworski J. (2007). TIMP-1 abolishes MMP-9-dependent long-lasting long-term potentiation in the prefrontal cortex. *Biological Psychiatry*.

[61] Knapska E., Lioudyno V., Kiryk A. (2013). Reward learning requires activity of matrix metalloproteinase-9 in the central amygdala. *Journal of Neuroscience*.

[62] Verslegers M., Lemmens K., Van Hove I., Moons L. (2013). Matrix metalloproteinase-2 and -9 as promising benefactors in development, plasticity and repair of the nervous system. *Progress in Neurobiology*.

[63] Ethell I. M., Ethell D. W. (2007). Matrix metalloproteinases in brain development and remodeling: synaptic functions and targets. *Journal of Neuroscience Research*.

[64] Klein T., Bischoff R. (2011). Physiology and pathophysiology of matrix metalloproteases. *Amino Acids*.

[65] Larsen P. H., DaSilva A. G., Conant K., Yong V. W. (2006). Myelin formation during development of the CNS is delayed in matrix metalloproteinase-9 and -12 null mice. *Journal of Neuroscience*.

[66] Mantuano E., Inoue G., Li X. Q. (2008). The hemopexin domain of matrix metalloproteinase-9 activates cell signaling and promotes migration of Schwann cells by binding to low-density lipoprotein receptor-related protein. *The Journal of Neuroscience*.

[67] Yamauchi K., Yamauchi T., Mantuano E. (2013). Low-density lipoprotein receptor related protein-1 (LRP1)-dependent cell signaling promotes neurotrophic activity in embryonic sensory neurons. *PLoS ONE*.

[68] Bruno M. A., Cuello A. C. (2006). Activity-dependent release of precursor nerve growth factor, conversion to mature nerve growth factor, and its degradation by a protease cascade. *Proceedings of the National Academy of Sciences of the United States of America*.

[69] Vaillant C., Meissirel C., Mutin M., Belin M.-F., Lund L. R., Thomasset N. (2003). MMP-9 deficiency affects axonal outgrowth, migration, and apoptosis in the developing cerebellum. *Molecular and Cellular Neuroscience*.

[70] Kessenbrock K., Plaks V., Werb Z. (2010). Matrix Metalloproteinases: regulators of the tumor microenvironment. *Cell*.

[71] Szklarczyk A., Lapinska J., Rylski M., McKay R. D., Kaczmarek L. (2002). Matrix metalloproteinase-9 undergoes expression and activation during dendritic remodeling in adult hippocampus. *The Journal of Neuroscience*.

[72] Scharfman H. E. (2002). Epilepsy as an example of neural plasticity. *Neuroscientist*.

[73] Chen Z.-L., Strickland S. (1997). Neuronal death in the hippocampus is promoted by plasmin-catalyzed degradation of laminin. *Cell*.

[74] Gottschall P. E., Yu X., Bing B. (1995). Increased production of gelatinase B (matrix metalloproteinase-9) and interleukin-6 by activated rat microglia in culture. *Journal of Neuroscience Research*.

[75] Van Lint P., Libert C. (2007). Chemokine and cytokine processing by matrix metalloproteinases and its effect on leukocyte migration and inflammation. *Journal of Leukocyte Biology*.

[76] De Bock M., Wang N., Decrock E., Bultynck G., Leybaert L. (2015). Intracellular cleavage of the Cx43 C-terminal domain by matrix-metalloproteases: a novel contributor to inflammation?. *Mediators of Inflammation*.

[77] Lee S.-R., Lo E. H. (2004). Induction of caspase-mediated cell death by matrix metalloproteinases in cerebral endothelial cells after hypoxia-reoxygenation. *Journal of Cerebral Blood Flow and Metabolism*.

[78] Lo E. H., Dalkara T., Moskowitz M. A. (2003). Mechanisms, challenges and opportunities in stroke. *Nature Reviews Neuroscience*.

[79] Mirrione M. M., Konomos D. K., Gravanis I. (2010). Microglial ablation and lipopolysaccharide preconditioning affects pilocarpine-induced seizures in mice. *Neurobiology of Disease*.

[80] Jabs R., Seifert G., Steinhäuser C. (2008). Astrocytic function and its alteration in the epileptic brain. *Epilepsia*.

[81] Binder D. K., Steinhäuser C. (2006). Functional changes in astroglial cells in epilepsy. *GLIA*.

[82] Vezzani A., Aronica E., Mazarati A., Pittman Q. J. (2013). Epilepsy and brain inflammation. *Experimental Neurology*.

[83] Fabene P. F., Bramanti P., Constantin G. (2010). The emerging role for chemokines in epilepsy. *Journal of Neuroimmunology*.

[84] Da Fonseca A. C. C., Matias D., Garcia C. (2014). The impact of microglial activation on blood-brain barrier in brain diseases. *Frontiers in Cellular Neuroscience*.

[85] Yu Q., Stamenkovic I. (2000). Cell surface-localized matrix metalloproteinase-9 proteolytically activates TGF-*β* and promotes tumor invasion and angiogenesis. *Genes and Development*.

[86] Van Vliet E. A., Araújo S. D. C., Redeker S., Van Schaik R., Aronica E., Gorter J. A. (2007). Blood-brain barrier leakage may lead to progression of temporal lobe epilepsy. *Brain*.

[87] Rosenberg G. A. (2009). Matrix metalloproteinases and their multiple roles in neurodegenerative diseases. *The Lancet Neurology*.

[88] Barr T. L., Latour L. L., Lee K.-Y. (2010). Blood-brain barrier disruption in humans is independently associated with increased matrix metalloproteinase-9. *Stroke*.

[89] Gidday J. M., Gasche Y. G., Copin J.-C. (2005). Leukocyte-derived matrix metalloproteinase-9 mediates blood-brain barrier breakdown and is proinflammatory after transient focal cerebral ischemia. *American Journal of Physiology—Heart and Circulatory Physiology*.

[90] Tsai H.-C., Chung L.-Y., Chen E.-R. (2008). Association of matrix metalloproteinase-9 and tissue inhibitors of metalloproteinase-4 in cerebrospinal fluid with blood-brain barrier dysfunction in patients with eosinophilic meningitis caused by Angiostrongylus cantonensis. *American Journal of Tropical Medicine and Hygiene*.

[91] Asahi M., Wang X., Mori T. (2001). Effects of matrix metalloproteinase-9 gene knock-out on the proteolysis of blood-brain barrier and white matter components after cerebral ischemia. *Journal of Neuroscience*.

[92] Reijerkerk A., Kooij G., van der Pol S. M. A., Khazen S., Dijkstra C. D., de Vries H. E. (2006). Diapedesis of monocytes is associated with MMP-mediated occludin disappearance in brain endothelial cells. *The FASEB Journal*.

[93] Zozulya A. L., Reinke E., Baiu D. C., Karman J., Sandor M., Fabry Z. (2007). Dendritic cell transmigration through brain microvessel endothelium is regulated by MIP-1*α* chemokine and matrix metalloproteinases. *Journal of Immunology*.

[94] Fabene P. F., Mora G. N., Martinello M. (2008). A role for leukocyte-endothelial adhesion mechanisms in epilepsy. *Nature Medicine*.

[95] Deem T. L., Cook-Mills J. M. (2004). Vascular cell adhesion molecule 1 (VCAM-1) activation of endothelial cell matrix metalloproteinases: role of reactive oxygen species. *Blood*.

[96] Agrawal S., Anderson P., Durbeej M. (2006). Dystroglycan is selectively cleaved at the parenchymal basement membrane at sites of leukocyte extravasation in experimental autoimmune encephalomyelitis. *The Journal of Experimental Medicine*.

[97] Van Den Steen P. E., Proost P., Wuyts A., Van Damme J., Opdenakker G. (2000). Neutrophil gelatinase B potentiates interleukin-8 tenfold by aminoterminal processing, whereas it degrades CTAP-III, PF-4, and GRO-*α* and leaves RANTES and MCP-2 intact. *Blood*.

[98] Van Den Steen P. E., Wuyts A., Husson S. J., Proost P., Van Damme J., Opdenakker G. (2003). Gelatinase B/MMP-9 and neutrophil collagenase/MMP-8 process the chemokines human GCP-2/CXCL6, ENA-78/CXCL5 and mouse GCP-2/LIX and modulate their physiological activities. *European Journal of Biochemistry*.

[99] Kim J. V., Kang S. S., Dustin M. L., McGavern D. B. (2009). Myelomonocytic cell recruitment causes fatal CNS vascular injury during acute viral meningitis. *Nature*.

[100] Fabene P. F., Laudanna C., Constantin G. (2013). Leukocyte trafficking mechanisms in epilepsy. *Molecular Immunology*.

[101] Gorkiewicz T., Szczuraszek K., Wyrembek P., Michaluk P., Kaczmarek L., Mozrzymas J. W. (2010). Matrix metalloproteinase-9 reversibly affects the time course of NMDA-induced currents in cultured rat hippocampal neurons. *Hippocampus*.

[102] Lin K.-T., Sloniowski S., Ethell D. W., Ethell I. M. (2008). Ephrin-B2-induced cleavage of EphB2 receptor is mediated by matrix metalloproteinases to trigger cell repulsion. *Journal of Biological Chemistry*.

[103] Lonskaya I., Partridge J., Lalchandani R. R. (2013). Soluble ICAM-5, a product of activity dependent proteolysis, increases mEPSC frequency and dendritic expression of GluA1. *PLOS ONE*.

[104] Jourquin J., Tremblay E., Décanis N. (2003). Neuronal activity-dependent increase of net matrix metalloproteinase activity is associated with MMP-9 neurotoxicity after kainate. *European Journal of Neuroscience*.

[105] Zhang J. W., Deb S., Gottschall P. E. (2000). Regional and age-related expression of gelatinases in the brains of young and old rats after treatment with kainic acid. *Neuroscience Letters*.

[106] Kim G. W., Kim H.-J., Cho K.-J., Kim H.-W., Cho Y.-J., Lee B. I. (2009). The role of MMP-9 in integrin-mediated hippocampal cell death after pilocarpine-induced status epilepticus. *Neurobiology of Disease*.

[107] Wang X., Lee S.-R., Arai K. (2003). Lipoprotein receptor-mediated induction of matrix metalloproteinase by tissue plasminogen activator. *Nature Medicine*.

[108] Gu Z., Kaul M., Yan B. (2002). S-nitrosylation of matrix metalloproteinases: signaling pathway to neuronal cell death. *Science*.

[109] Copin J.-C., Goodyear M.-C., Gidday J. M. (2005). Role of matrix metalloproteinases in apoptosis after transient focal cerebral ischemia in rats and mice. *European Journal of Neuroscience*.

[110] Konopacki F. A., Rylski M., Wilczek E. (2007). Synaptic localization of seizure-induced matrix metalloproteinase-9 mRNA. *Neuroscience*.

[111] Frantseva M. V., Perez Velazquez J. L., Tsoraklidis G. (2000). Oxidative stress is involved in seizure-induced neurodegeneration in the kindling model of epilepsy. *Neuroscience*.

[112] Hoehna Y., Uckermann O., Luksch H. (2012). Matrix metalloproteinase 9 regulates cell death following pilocarpine-induced seizures in the developing brain. *Neurobiology of Disease*.

[113] Takács E., Nyilas R., Szepesi Z. (2010). Matrix metalloproteinase-9 activity increased by two different types of epileptic seizures that do not induce neuronal death: a possible role in homeostatic synaptic plasticity. *Neurochemistry International*.

[114] Zahn R. K., Tolner E. A., Derst C., Gruber C., Veh R. W., Heinemann U. (2008). Reduced ictogenic potential of 4-aminopyridine in the perirhinal and entorhinal cortex of kainate-treated chronic epileptic rats. *Neurobiology of Disease*.

[115] Horch H. W., Krüttgen A., Portbury S. D., Katz L. C. (1999). Destabilization of cortical dendrites and spines by BDNF. *Neuron*.

[116] Giorgi O., Orlandi M., Geic M., Corda M. G. (1991). Mk-801 prevents the decrease in 35S-TBPS binding in the rat cerebral cortex induced by pentylenetetrazol kindling. *Brain Research Bulletin*.

[117] Murase S., Lantz C. L., Kim E. (2015). Matrix metalloproteinase-9 regulates neuronal circuit development and excitability. *Molecular Neurobiology*.

[118] Mun-Bryce S., Rosenberg G. A. (1998). Gelatinase B modulates selective opening of the blood-brain barrier during inflammation. *American Journal of Physiology*.

[119] Rosell A., Cuadrado E., Ortega-Aznar A., Hernández-Guillamon M., Lo E. H., Montaner J. (2008). MMP-9-positive neutrophil infiltration is associated to blood-brain barrier breakdown and basal lamina type IV collagen degradation during hemorrhagic transformation after human ischemic stroke. *Stroke*.

[120] Friedman A., Kaufer D., Heinemann U. (2009). Blood–brain barrier breakdown-inducing astrocytic transformation: Novel targets for the prevention of epilepsy. *Epilepsy Research*.

[121] Lu C., Li J., Sun W. (2010). Elevated plasma S100B concentration is associated with mesial temporal lobe epilepsy in Han Chinese: A Case-control Study. *Neuroscience Letters*.

[122] Kwan P., Arzimanoglou A., Berg A. T. (2010). Definition of drug resistant epilepsy: consensus proposal by the ad hoc Task Force of the ILAE Commission on Therapeutic Strategies. *Epilepsia*.

[123] Wiebe S., Jetté N. (2012). Epilepsy surgery utilization: who, when, where, and why?. *Current Opinion in Neurology*.

[124] Curia G., Lucchi C., Vinet J. (2014). Pathophysiogenesis of mesial temporal lobe epilepsy: is prevention of damage antiepileptogenic?. *Current Medicinal Chemistry*.

[125] Aliashkevich A. F., Yilmazer-Hanke D., Van Roost D., Mundhenk B., Schramm J., Blümcke I. (2003). Cellular pathology of amygdala neurons in human temporal lobe epilepsy. *Acta Neuropathologica*.

[126] Yilmazer-Hanke D. M., Wolf H. K., Schramm J., Elger C. E., Wiestler O. D., Blümcke I. (2000). Subregional pathology of the amygdala complex and entorhinal region in surgical specimens from patients with pharmacoresistant temporal lobe epilepsy. *Journal of Neuropathology and Experimental Neurology*.

[127] Engel J. (2001). Mesial temporal lobe epilepsy: what have we learned?. *Neuroscientist*.

[128] Czech T., Yang J.-W., Csaszar E., Kappler J., Baumgartner C., Lubec G. (2004). Reduction of hippocampal collapsin response mediated protein-2 in patients with mesial temporal lobe epilepsy. *Neurochemical Research*.

[129] Crespel A., Coubes P., Rousset M.-C. (2002). Inflammatory reactions in human medial temporal lobe epilepsy with hippocampal sclerosis. *Brain Research*.

[130] Boer K., Spliet W. G. M., van Rijen P. C., Redeker S., Troost D., Aronica E. (2006). Evidence of activated microglia in focal cortical dysplasia. *Journal of Neuroimmunology*.

[131] Maldonado M., Baybis M., Newman D. (2003). Expression of ICAM-1, TNF-*α*, NF*κ*B, and MAP kinase in tubers of the tuberous sclerosis complex. *Neurobiology of Disease*.

[132] Li S., Yu S., Zhang C. (2012). Increased expression of matrix metalloproteinase 9 in cortical lesions from patients with focal cortical dysplasia type IIb and tuberous sclerosis complex. *Brain Research*.

[133] Suenaga N., Ichiyama T., Kubota M., Isumi H., Tohyama J., Furukawa S. (2008). Roles of matrix metalloproteinase-9 and tissue inhibitors of metalloproteinases 1 in acute encephalopathy following prolonged febrile seizures. *Journal of the Neurological Sciences*.

[134] Brew K., Dinakarpandian D., Nagase H. (2000). Tissue inhibitors of metalloproteinases: evolution, structure and function. *Biochimica et Biophysica Acta (BBA)—Protein Structure and Molecular Enzymology*.

[135] Kittaka S., Hasegawa S., Ito Y. (2014). Serum levels of matrix metalloproteinase-9 and tissue inhibitor of metalloproteinases-1 in human herpesvirus-6-infected infants with or without febrile seizures. *Journal of Infection and Chemotherapy*.

[136] Suriadi M. M., Takahashi Y., Nishimura S., Tsunogae H., Inoue Y. (2012). Dysfunction of blood-brain barrier in epileptic patients after acute encephalitis. *Journal of Epileptology*.

[137] Ichiyama T., Morishima T., Kajimoto M., Matsushige T., Matsubara T., Furukawa S. (2007). Matrix metalloproteinase-9 and tissue inhibitors of metalloproteinases 1 in influenza-associated encephalopathy. *The Pediatric Infectious Disease Journal*.

[138] Quirico-Santos T., Nascimento Mello A., Casimiro Gomes A., De Carvalho L. P., De Souza J. M., Alves-Leon S. (2013). Increased metalloprotease activity in the epileptogenic lesion—lobectomy reduces metalloprotease activity and urokinase-type uPAR circulating levels. *Brain Research*.

[139] Ainiala H., Hietaharju A., Dastidar P. (2004). Increased serum matrix metalloproteinase 9 levels in systemic lupus erythematosus patients with neuropsychiatric manifestations and brain magnetic resonance imaging abnormalities. *Arthritis and Rheumatism*.

[140] Cudna A., Kurkowska-Jastrzębska I. (2014). Blood-brain barrier markers after acute epileptic seizures. *Journal of Neuroimmunology*.

[141] Cudna A., Jopowicz A., Mierzejewski P., Kurkowska-Jastrzębska I. (2017). Serum metalloproteinase 9 levels increase after generalized tonic-clonic seizures. *Epilepsy Research*.

[142] Leppert D., Leib S. L., Grygar C., Miller K. M., Schaad U. B., Holländer G. A. (2000). Matrix metalloproteinase (MMP)-8 and MMP-9 in cerebrospinal fluid during bacterial meningitis: association with blood-brain barrier damage and neurological sequelae. *Clinical Infectious Diseases*.

[143] Shahar E., Attias U., Savulescu D., Genizin J., Gavish M., Nagler R. (2014). Oxidative stress, metalloproteinase and LDH in children with intractable and non-intractable epilepsy as reflected in salivary analysis. *Epilepsy Research*.

[144] Sienkiewicz-Jarosz H., Ryglewicz D. (2007). Evaluation of biomarkers in stroke. *Problemy Interdyscyplinarne*.

[145] Lynch J. R., Blessing R., White W. D., Grocott H. P., Newman M. F., Laskowitz D. T. (2004). Novel diagnostic test for acute stroke. *Stroke*.

[146] Montaner J., Mendioroz M., Ribó M. (2011). A panel of biomarkers including caspase-3 and d-dimer may differentiate acute stroke from stroke-mimicking conditions in the emergency department. *Journal of Internal Medicine*.

[147] Šímová J., Škvor J., Slovák D., Mazura I., Zvárová J. (2013). Serum levels of matrix metalloproteinases 2 and 9 in patients with acute myocardial infarction. *Folia Biologica*.

[148] Galliera E., Tacchini L., Romanelli M. M. C. (2015). Matrix metalloproteinases as biomarkers of disease: updates and new insights. *Clinical Chemistry and Laboratory Medicine*.

[149] Leppert D., Lindberg R. L. P., Kappos L., Leib S. L. (2001). Matrix metalloproteinases: multifunctional effectors of inflammation in multiple sclerosis and bacterial meningitis. *Brain Research Reviews*.

[150] Groblewska M., Siewko M., Mroczko B., Szmitkowski M. (2012). The role of matrix metalloproteinases (MMPs) and their inhibitors (TIMPs) in the development of esophageal cancer. *Folia Histochemica et Cytobiologica*.

[151] Roy R., Yang J., Moses M. A. (2009). Matrix metalloproteinases as novel biomarkers and potential therapeutic targets in human cancer. *Journal of Clinical Oncology*.

